# Estimating Socio-Economic Status for Alzheimer’s Disease Trials

**DOI:** 10.14283/jpad.2024.88

**Published:** 2024-05-07

**Authors:** Dorene M. Rentz, J. D. Grill, D. P. Molina-Henry, G. A. Jicha, M. S. Rafii, A. Liu, R. A. Sperling, P. S. Aisen, R. Raman

**Affiliations:** 1 Departments of Neurology, Massachusetts General Hospital, Brigham and Women’s Hospital, Harvard Medical School, 60 Fenwood Road, Boston, MA 02115 USA; 2 Institute for Memory Impairments and Neurological Disorders, Department of Psychiatry & Human Behavior, Department of Neurobiology & Behavior, University of California, Irvine, CA 92697 USA; 3 Alzheimer’s Therapeutic Research Institute, Keck School of Medicine, University of Southern California, San Diego, CA 92121 USA; 4 Department of Neurology and the Sanders-Brown Center on Aging, University of Kentucky College of Medicine, Lexington, KY 40536 USA

**Keywords:** Socioeconomic status, socioeconomic indicators, clinical trials

## Abstract

**Introduction:**

Metrics of a participant’s socioeconomic status (SES) are not routinely collected or standardized in clinical trials. This omission limits the ability to evaluate the generalizability of trial results and restricts clinicians from confidently interpreting the efficacy of new treatments across important sub-populations.

**Methods:**

We adapted an SES measure of social disparity; the Hollingshead Two Factor Index of Social Position, which combines education and occupation into a single metric. We modernized the 1965 occupations to reflect the 2017 careers tabulated by the US Bureau of Labor Statistics. We currently use this adapted measure in Alzheimer’s Clinical Trials Consortium studies.

**Results:**

We present the revised table of occupations. We found that the collection of SES data using the modified Hollingshead was feasible in a multi-site clinical trial and scores were distributed across all SES strata.

**Discussion:**

The modified Hollingshead provides a standardized method for collecting SES information, enabling data aggregation, monitoring, and reporting.

## Introduction

**E**thnic and racially minoritized groups, as well as individuals who come from socially underserved populations, are historically underrepresented in clinical trials of Alzheimer’s Disease (AD) (1) but carry a disproportionately increased risk of cognitive decline and dementia (2). The National Institute of Aging (NIA) and the Food and Drug Administration (FDA) have recognized this disparity and mandate the enrollment into clinical trials of an inclusive sample of individuals from underserved groups, including racial and ethnic underrepresented populations (URP’s). While race and ethnicity data are now routinely collected in trials, additional socioeconomic (SES) information about participants is not frequently collected. Such information may include employment type, neighborhood classification (rural/urban, advantage/disadvantage level) and other environmental/occupational attributes. Even if collected, these metrics are infrequently standardized in a manner to permit effective and improved data quality, usability, and integration across studies. This lack of knowledge and standardization about clinical trial participants can limit the generalizability of clinical trial data (1, 3) and restricts clinicians from confidently interpreting the efficacy of new treatments across all populations. Knowledge of SES data would also inform on observed health disparity, as well as determinants of disease and disease course, and provide data useful for health economics research and health policy (4).

Our goal was to standardize the collection of SES data for inclusion into our NIH funded Alzheimer’s Disease Clinical Trial Consortium (ACTC) of studies as part of a minimal data set (MDS). We explored SES scales that were brief, validated and contained SES constructs commonly viewed as important indicators related to SES in US and non-US countries. Our search included scales developed by August B. Hollingshead at Yale University; i.e., The Two Factor Index of Social Position (5) developed in 1965, which measures social status based on education and occupation and continues to be used in the Harvard Aging Brain Study, an NIH funded longitudinal cohort of older individuals (6). We also examined Hollingshead’s 1975 version of the Four Factor Index of Social Position (7) that includes marital status, retired/employed status, educational attainment and occupational prestige. In reviewing the literature, additional SES variables exploring inequalities around the world were surprisingly similar. The widely used Kuppuswamy SES scale developed in India in 1976 (8) comprises a composite score of education and occupation of the Family Head along with family income per month. A population study in France used education, occupation and income to explore inequalities in dementia risk (9). Education and occupational class were used in 22 countries in Europe to explore health outcomes (10), and education, income and employment status were used in a prospective study from the UK Biobank to assess socioeconomic factors in early and late-onset dementia (11). Finally, in 2012, the NIH National Committee on Vital Health Statistics (NCVHS) published a minimum set of questions for measuring SES including education, occupation, total income and family size www.ncvhs.hhs.gov. Some of the scales presented above, as well as other scales used in population health (12) were rejected for the ACTC MDS because they would be cumbersome to implement at clinical trial sites, create missing or duplicate data (i.e., marital status, retired or employed), or involve the collection of a multitude of socioeconomic indicators including family income, education, early childhood experiences, physical environment and neighborhood residence that may seem intrusive or irrelevant for our purposes.

Based on this initial research, we chose to modify the Hollingshead Two Factor Index of Social Position because it required a minimal set of questions that could be transformed into a single SES metric for monitoring recruitment and facilitating data aggregation. For our purposes, the Hollingshead Occupational Employment Status scale was modernized with the on-line 2017 table of occupations from the Bureau of Labor Statistics (13), since many of the occupations used in the 1965 version were antiquated. This modified Hollingshead, was added to the NIA funded ACTC’s MDS, for use in all ACTC studies and is now being collected during the screening visit in three ongoing ACTC clinical trials, namely, the AHEAD program (aheadstudy.org, NCT04468659), and two Phase 2 studies i.e., START (start-study. org, NCT05531656) and the LiBBY trial, (libbystudy. org, NCT05644262). We anticipate that this modified Hollingshead composite will help provide a more inclusive picture of our clinical trial participants. Here, we describe how it was adapted for the ACTC MDS and show how the scale operates in the ongoing AHEAD study.

## Methods

### The Two Factor Index of Social Position

The 1965 version of the Hollingshead Two Factor Index of Social Position (5) ranks a list of occupations from professionals/ higher executives and business owners to less technical professions such as skilled labor employees and unskilled workers on a 7-point scale (See Table 1). Educational attainment is stratified from professional /post graduate degrees to those with less than 7 years of education (see Table 1). The Two Factor Index score is calculated with factor weights based on rank of occupation and level of education as follows: (occupational rank X 7) + (educational rank X 4). This produces a single composite score of social position from 11 to 77 with lower scores indicating higher SES (11–17) and higher scores indicating lower SES (63–77). Intermediate rankings from higher to lower SES included scores ranging from 18–31, 32–47, and 48–62. This single combined metric eliminates the bias of those who did not have opportunities for higher education but occupationally achieved advanced SES stature.

**Table 1:** Ranking of occupations and education on the modified Hollingshead

**Occupational Ranking**		
Major Professionals/ Higher Executives/ Proprietors of Large Concerns		
Architects	1	
Aeronautical Engineers	1	
Astronauts		
Bank Presidents	1	
Business Owners	1	
Certified Public Accountant	1	
Chief Executive/ CEO, CFO, COO	1	
Clergy	1	
Commissioned Officers in the Military	1	
Dentists	1	
Economists	1	
Engineers/ master’s level and above	1	
Geneticists	1	
Lawyers/ Judges	1	
Major Contractors	1	
Physicians	1	
Professor/ University Teachers	1	
Psychologists	1	
Research Scientists/ Statisticians /PhD	1	
Veterinarians	1	
VP of Large Business	1	
Lesser Professionals/ Business Managers of Medium-Sized Businesses		
Accountants		
Advertising Executives	2	
Branch Managers	2	
Building Contractors	2	
Business Managers	2	
Chiropractors	2	
Computer Programmer	2	
Database Developer/ Administrator	2	
Engineers- no advanced degree	2	
Executive Managers	2	
Farm Owners	2	
Furniture Business	2	
Government Officials	2	
Jewelers	2	
Labor Relations Consultant	2	
Librarians	2	
Manufacturing Owners	2	
Musicians	2	
Nurses	2	
Office Managers	2	
Opticians	2	
Personnel Managers	2	
Pharmacists	2	
Police Chief/ Sheriff	2	
Postmaster	2	
Production Managers/ TV/ Radio	2	
Public Health Officers- non-MD	2	
Purchasing Managers	2	
Real Estate Brokers	2	
Sales Engineers	2	
Sales Managers	2	
Social Workers	2	
Teachers/ Elementary & High School	2	
Theatre Owners	2	
Administrative Personnel, Small Business Owners, Minor Professionals		
Actors	3	
Administrative Assistants	3	
Advertising Agents	3	
Artists	3	
Bakers	3	
Beauty Shop Owners	3	
Chefs		
Chief Clerks	3	
Clergy- not professionally trained	3	
Court Reporters	3	
Credit Managers	3	
Department Store Manager	3	
Deputy Sheriffs	3	
Dispatchers	3	
Farmers	3	
Florists	3	
Funeral Directors	3	
Government Officials	3	
Insurance Agents	3	
Laboratory Assistants	3	
Landscape Planners	3	
Military NCO/ Sgts	3	
Morticians	3	
Newspaper / TV Reporters	3	
Oral Hygienists	3	
Photographers	3	
Piano Teachers	3	
Plumbers	3	
Radio/ TV Announcers	3	
Real Estate Agents	3	
Research Assistants	3	
Restaurant Owners	3	
Sales Representatives	3	
Service Managers	3	
Small Business Owners	3	
Store Managers	3	
Surveyors	3	
Title Searchers	3	
Tool Designers	3	
Traffic Managers	3	
Travel Agents	3	
Yard Masters/ Railroad	3	
Clerical and Sales Workers, Technicians, Owners of Little Businesses		
Bank Tellers	4	
Bill Collectors	4	
Bookkeepers	4	
Claims Examiners	4	
Dental Technician	4	
Draftsman	4	
Driving Teacher	4	
Factory Supervisors	4	
Flower Shop Worker	4	
Human Resource Interviewer	4	
Laboratory Technicians	4	
Newsstand Operator	4	
Post Office Clerk	4	
Railroad Conductors	4	
Railroad Train Engineers	4	
Route Managers	4	
Salesclerks	4	
Secretaries	4	
Shipping Clerks	4	
Tailor	4	
Tax Clerks	4	
Telephone Company Worker	4	
Timekeepers	4	
Truck Dispatchers	4	
Utility Worker	4	
Warehouse Clerks	4	
Window Store Trimmers	4	
Skilled Manual Employees		
Auto Body Repairs	5	
Barbers	5	
Blacksmiths	5	
Boiler Repairmen	5	
Bookbinders	5	
Brewers	5	
Bulldozer Operators	5	
Cabinet Makers	5	
Carpenters	5	
Cement Layers/ Finishers	5	
Cheese Makers	5	
Construction Foreman	5	
Diemakers	5	
Electricians	5	
Engravers	5	
Exterminators	5	
Firemen	5	
Gardeners/ Landscapers	5	
Glassblowers	5	
Glaziers	5	
Gun Smiths	5	
Hair Stylists	5	
Homemaker	5	
Home Repairmen	5	
Kitchen Workers/ Cooks	5	
Locksmiths	5	
Machinists	5	
Mailmen	5	
Maintenance Foreman	5	
Masons	5	
Mechanics	5	
Millwrights	5	
Painters	5	
Paperhangers	5	
Patrolmen	5	
Piano Builders	5	
Piano Tuners	5	
Plumbers	5	
Policemen	5	
Postmen	5	
Printers	5	
Radio/ TV Maintenance	5	
Railroad Brakeman	5	
Sheetmetal Workers	5	
Shoe Repairmen	5	
Tile Layers	5	
Tool Makers	5	
Upholsterers	5	
Utility Linemen	5	
Watchmakers	5	
Weavers	5	
Welders	5	
Machine Operators and Semiskilled Employees		
Apprentices (Electrician/Printers/etc.)	6	
Assembly Line Workers	6	
Bartenders	6	
Building Superintendent	6	
Bus Drivers	6	
Cab/ Taxi Drivers	6	
Cashiers	6	
Cooks- Short Order	6	
Delivery men	6	
Dry Cleaning Pressers	6	
Elevator Operators	6	
Enlisted Military Personnel	6	
Factory Machine Operators	6	
Factory Workers	6	
Foundry Workers	6	
Garage and Gas Station Assistants	6	
Greenhouse Workers	6	
Guards, Security Watchmen	6	
Machine Operators and semiskilled	6	
Meat Cutters/ Packers	6	
Meter Readers	6	
Oil Delivery Men	6	
Practical Nurses	6	
Pump Operators	6	
Roofers	6	
Seamstresses	6	
Signal Men- Railroad	6	
Trucker Driver	6	
Waiters/ Waitresses	6	
Wine Bottlers	6	
Wood Workers	6	
Wrappers- Stores and Factories	6	
Unskilled Employees		
Amusement Park Workers	7	
Cafeteria Workers	7	
Car Cleaners	7	
Construction Laborers	7	
Deck Hands	7	
Domestics	7	
Farm Helpers	7	
Fishermen	7	
Freight Handlers	7	
Hospital Housekeepers	7	
Janitors	7	
Junk/ Recycle Sorters	7	
Laundry Workers	7	
Messengers	7	
Peddlers	7	
Porters	7	
Roofer Laborers	7	
Shoe Shiners	7	
Stagehands	7	
Stock Handlers	7	
Street Cleaners	7	
Unskilled Factory Workers	7	
Unspecified Laborers	7	
Window Cleaners	7	
Woodchoppers	7	
**Educational Ranking**		
**Category**	**Years of Education**	**Score**
Professional (MA, MS, ME, MD, PhD, LLD, JD)	18 Years or more	1
Four-year college graduate (AB, BA, BS, BM)	16 to 17 years	2
One to three years college (business schools)	13 to 15 years	3
High school graduate	12 years	4
Ten to eleven years of school	10 to 11 years	5
Seven to nine years of school	7 to 9 years	6
Less than seven years of school	Less than 7 years	7

Since the development of the Two Factor Index in 1965, the importance and type of occupations have dramatically changed societally. We modernized the Two Factor Index with current occupations listed in the on-line 2017 table from the Bureau of Labor Statistics (13). We felt this was necessary as the original list of occupations did not contain modern advancements in technology that have significantly shifted common job classifications. Major changes that differed from the original Hollingshead scale in 1965 but were included in the 2017 table from the Bureau of Labor Statistics included:

1. The addition of occupations such as computer-related jobs, database developers/ database administrators, aeronautical engineers, astronauts, geneticists, statisticians in advanced methods (AI, ML, etc.).
2. Occupations, such as plumbers and farmers were ranked higher because they require more advanced computerized skills.
3. Homemakers were ranked higher today than in 1965.
4. Occupations that were eliminated from the original Hollingshead scale were professions such as railroad car cleaners, grave diggers, shirt folders, and teletype operators, etc.

Of the 227 occupations listed, we left approximately 75% of the occupational rankings the same as they were considered still relevant today.

### Participant Data

**Figure 1:**
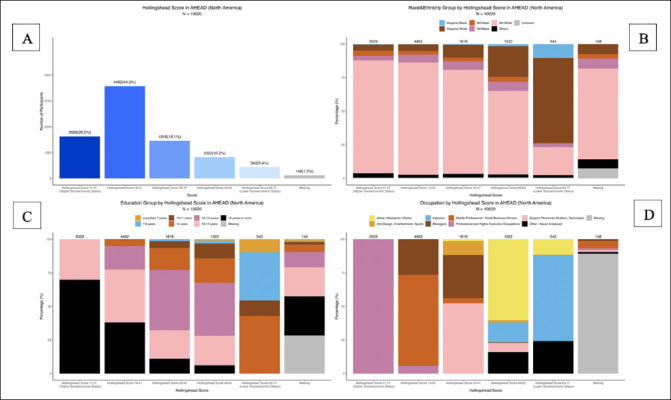
Distribution of the AHEAD study screening Hollingshead score at 51% study enrollment (n=10020) A: Distribution of the Hollingshead scores grouped by socio-economic status (SES) categories. B: Stacked bar plot of the distribution of the Hollingshead score across the combined race and ethnicity categories. C: Stacked bar plot of the distribution of the Hollingshead score by education group categories. D: Stacked bar plot of the distribution of the Hollingshead score by occupation group categories.

This modified Hollingshead scale was implemented as part of the ACTC MDS in the first ACTC trial, the AHEAD study. AHEAD is a secondary prevention trial funded by the NIA, Eisai and several philanthropic organizations, and conducted as a public-private partnership at 75 US and Canadian sites between ACTC and Eisai. Sites were asked to administer the modernized Hollingshead version that incorporated the 2017 occupations. The clinical trial study coordinators asked participants their education and occupation during the first screening visit of the AHEAD study. Education is collected as the level of education achieved, while occupation is collected using the major categories listed in Table 1. The education categorization and the computation of the actual Hollingshead Score presented Figure 1 was tabulated by the ACTC biostatistics unit. The data reported in this work include only screened participants from North American sites from study start to 08-2023 (51% randomization complete). This preliminary analysis was designed to evaluate the performance of this scale, at this juncture, to ensure that the scale was functioning as expected. Participants ranged in age from 55–80 (n=10,020).

## Results

Figure 1A shows the distribution of the Hollingshead two factor composite based on occupation and education across all social strata in the AHEAD study. The skewness is toward middle (44.5%) rather than higher SES (20.2%) but the distribution is spread across all categories including managers, support personnel and technicians (18.1%), aides, clerks and skilled laborers (10.2%) and unskilled workers (5.4%) (Figure 1D). Figure 1C maps the distribution of education alone across the same SES strata. An examination of both Figures 1A and 1C indicates that those with higher levels of education, i.e., college and advanced degrees are employed at all levels of the SES strata suggesting that education alone does not accurately represent occupational status. There are some individuals with a high school education who are executives and business owners and some with higher degrees who have never been employed. Figure 1B shows the SES distribution across race and ethnicity. While there is a larger proportion of non-Hispanic whites in the sample, the distribution of race and ethnicity is across all social strata rather than skewing toward higher SES. Non-Hispanic Blacks and Asians are equally distributed but Hispanic Whites and Blacks tend to fall in the lower socioeconomic group. Most importantly, the study data was largely complete (98.5%) with minimal missing data (1.5%), suggesting that data collection was feasible across study sites and all demographic groups.

## Discussion

We modernized the Hollingshead Two Factor Index of Social Position with occupations from the 2017 US Bureau of Labor Statistics. Our goal was to standardize the collection of socioeconomic metrics in addition to the routine data collected on race and ethnicity to ensure the generalizability of data stemming from clinical trials in dementia prevention and treatment. We anticipated that by more fully characterizing SES, the modified Hollingshead two-factor data would help resolve some of the discrepancies in data collection regarding high or low education, white- or blue-collar jobs and urban vs. rural or unskilled occupations. This knowledge and standardization about SES in our clinical trial participants is designed to aid in the generalizability of clinical trial data (1, 3) and help clinicians confidently interpret the efficacy of new treatments across disparate participants and populations. In the future, this more detailed estimate of SES attainment might serve to better explain risk of future cognitive decline, determinants of disease course and/or response to therapeutic interventions. It will also be important to determine how these SES variables will translate into other countries and cultures and whether they will operate in a similar fashion.

We found that collecting SES data using the modernized Hollingshead was feasible with only 1.5% missing data in a total of 10,020 participants from the AHEAD study screening visit. The value of a single SES composite ranks social position without the bias that may occur when using education alone, particularly in older, and/or diverse individuals who may not have had the advantage or opportunity for higher education. This was evident in the graph that demonstrates that education level alone does not always map onto occupational advancement. We also discovered that race and ethnicity was equally distributed across all social strata rather than skewing toward higher SES. There was a preponderance of Hispanic Whites and Blacks in the lower socioeconomic group, which was likely due to the location of clinical trial sites who have individuals in lower socioeconomic conditions or other factors inherent in our socioeconomic and political systems. These findings are important and suggest that a standardized composite, such as the modified Hollingshead can permit the evaluation of recruitment across SES more accurately and can allow for the necessary adjustment of recruitment strategies to obtain inclusive samples in ACTC trials. In addition, the modernized Hollingshead may serve to disambiguate the influence of social determinants of health versus potential biologic or genetic effects often attributed to race and/or ethnicity.

While we chose the Two Factor Index of Social Position, for all the features mentioned above, we recognize that the measurement and standardization of SES is highly complex and challenging. Interestingly, the SES variables used in global epidemiological studies were comparable to what was recommend by the NIH National Committee on Vital Health Statistics (NCVHS) (i.e., education, occupation, income and family size/household composition) but in contrast to the Hollingshead, are not presented as a single composite, which is more suitable for data aggregation and interpreting the generalizability of clinical trial results.

While income was considered a key variable in the NCVHS recommendation and included in many global epidemiological studies, there were several reasons why we rejected this variable for our own purposes. For example, a wealth/ income index used in 90 countries for measuring malnutrition in children (14) was found to be inappropriate because the poorest wealth in some countries may be the highest wealth in others. This corresponds to the disparate cost of living estimates affecting SES across geographic regions in the United States. We also felt that older participants would perceive this inquiry as intrusive and might result in misclassification or significant missing data. In addition, we were unable to identify approaches to standardize income as a sole measure of SES given the numerous sources of income received by individuals in the United States (i.e., social security, property, wages, retirement income, public assistance, total net worth, etc.) and whether such metrics would utilize highest or current income.

Occupation was considered important in our choice of an SES scale, but it can also be challenging to collect since occupation is not fixed during an individual’s lifetime; rather, it varies by levels of occupation (current or highest level), length of an occupation (longest occupation), or time spent on the job, etc. We opted to go for the highest level of occupation during one’s lifetime as we wanted to capture the maximum level of occupational status achieved and avoid retirement as a collected variable. In addition, the modified Hollingshead provides a way to classify a myriad of occupations with a single ranking, simplifying this complex category for more accurate data aggregation. Other health disparity studies include workplace exposures and reasons for not working but this seemed less relevant in our older clinical trial participants (15).

Consistent with the NCVHS recommendation and other global studies, we agreed that education was critical in SES. The modified Hollingshead provides a simplified method for classifying this variable, eliminating the error of tabulating total years spent in school rather than the level of education achieved. We chose not to collect data on household size in the MDS because we are already collecting relevant information about study partner demographics. In the Anti-Amyloid in Asymptomatic Alzheimer’s Disease (A4) study, we found important differences in study-partner dyads (i.e., spouse vs. child vs. grandchild vs. friend), particularly among racial and ethnic groups (3). Thus, size of household seemed less relevant than the study-partner demographics already being collected.

Essentially, our decision to incorporate the modified Hollingshead into the ACTC MDS aimed to establish a standardize SES measure with a concise set of questions that would enhance data quality, usability, and cross-study integration. While we acknowledge the modified Hollingshead’s limitations, such as absence of variables like income, neighborhood type (rural/urban) or advantage/disadvantage level (ADI), it offers a straightforward means to gauge social standing, aiding the generalizability of clinical trial data. We will actively monitor the modified Hollingshead’s implementation in ACTC trials and determine if the distribution of SES differs between the AHEAD screening participants and the ultimate randomized population in AHEAD. We are also monitoring SES data collection in other symptomatic ACTC studies and will determine if there is a difference in SES status between asymptomatic and symptomatic groups. Nonetheless, the findings presented here suggest that the modified two-factor Hollingshead for collecting SES data in this manner holds promise for yielding thorough and accurate estimates, resulting in the most comprehensive data possible.

### Limitations and Future Directions

While we used the US Bureau of Labor Statistics to define current occupations, we did not rank the occupations with scientific rigor. A more rigorous approach would have been to hold a focus or consensus group to determine occupational ranking. Due to substantial efforts to increase the diversity of participants screening for ACTC clinical trials, the AHEAD screening cohort appears more inclusive of the population at risk for Alzheimer’s disease dementia when compared to previously enrolled trials. However, it is unclear if this will persist in the randomized population, and we intend to monitor SES throughout the trial.

In the future, we anticipate that this additional SES data, collected in our global clinical trials, could facilitate the development of a conceptual framework for exploring mechanistic pathways or causal relationships between SES, cognition and treatment outcomes. We also anticipate that this additional SES data could be used to assess health disparities and the role that education, occupation and resilience may have on disease progression. Finally, it will be important to determine whether the SES data collected in US ACTC trials, can be applicable to AD clinical trials in other countries and whether this data functions differently across cultures.

## Conclusions

Establishing standardized metrics for collecting SES data in clinical trials can facilitate the interpretation of clinical trial findings across all populations. The modified Hollingshead utilized by the ACTC as part of the ACTC MDS provides such a metric. Further validation of the modernized occupations, applicability in other countries and cultures, and its useability as an outcome measure is on-going.

### Research context

1. Systematic review: Metrics about race and ethnicity are routinely collected in Alzheimer disease (AD) clinical trials. However, other demographic information that would inform on determinants of disease or disease course, as well as facilitate the generalizability of clinical trial results across important sub-populations are not often collected. We searched the literature for SES scales that could be incorporated into the NIH funded Alzheimer’s Clinical Trials Consortium Minimal Data Set (ACTC MDS) to provide a standardized method for enabling data aggregation, monitoring and reporting.
2. Interpretation: This paper serves as a resource for implementing a modified Hollingshead Two Factor Index of Social Position with current modern occupations. We demonstrate excellent feasibility and distribution across all SES strata with minimal data loss in a multi-site clinical trial.
3. Future directions: Further work is needed to validate the revised list of modern occupations against the older list of occupations and to measure its utility and impact on trial outcomes world-wide.

## Notes

*Ethical standards:* All participants provided central IRB-approved informed consent prior to data collection.
